# A GAP that Divides

**DOI:** 10.12688/f1000research.12064.1

**Published:** 2017-10-02

**Authors:** Angika Basant, Michael Glotzer

**Affiliations:** 1 The Francis Crick Institute, London, UK; 2 Department of Molecular Genetics and Cell Biology, University of Chicago, Chicago, Illinois, USA

**Keywords:** GAP proteins, RhoA, cytokinesis

## Abstract

Cytokinesis in metazoan cells is mediated by an actomyosin-based contractile ring that assembles in response to activation of the small GTPase RhoA. The guanine nucleotide exchange factor that activates RhoA during cytokinesis, ECT-2, is highly regulated. In most metazoan cells, with the notable exception of the early *Caenorhabditis elegans* embryo, RhoA activation and furrow ingression require the centralspindlin complex. This exception is due to the existence of a parallel pathway for RhoA activation in *C. elegans*. Centralspindlin contains CYK-4 which contains a predicted Rho family GTPase-activating protein (GAP) domain. The function of this domain has been the subject of considerable debate. Some publications suggest that the GAP domain promotes RhoA activation (for example, Zhang and Glotzer, 2015; Loria, Longhini and Glotzer, 2012), whereas others suggest that it functions to inactivate the GTPase Rac1 (for example, Zhuravlev *et al*., 2017). Here, we review the mechanisms underlying RhoA activation during cytokinesis, primarily focusing on data in *C. elegans.* We highlight the importance of considering the parallel pathway for RhoA activation and detailed analyses of *cyk-4* mutant phenotypes when evaluating the role of the GAP domain of CYK-4.

## Activation of RhoA by the RhoGEF ECT-2 directs furrow formation

Shortly after mitotic chromosomes separate, the small GTPase, RhoA, accumulates in its active form at the equatorial plasma membrane of animal cells ^1– 4^. This pool of RhoA-GTP is necessary ^5– 7^ and sufficient ^8^ for the assembly of an actomyosin-driven furrow that partitions a cell into two daughter cells.

As cytokinesis occurs at a specific site at a precise time, RhoA activation is necessarily highly regulated. In metazoa, RhoA activation during cytokinesis is mediated by a Rho guanine nucleotide exchange factor (RhoGEF), ECT-2, which plays a well-conserved role in activating RhoA during cytokinesis ^9– 15^. However, ECT-2 itself is highly regulated; its activity is suppressed by autoinhibition and phosphorylation, and it is activated by protein-protein interactions and dephosphorylation. Here, we discuss recent progress in defining the role of an atypical Rho GTPase-activating protein (GAP), CYK-4, protein in RhoA activation during cytokinesis with a focus on the early *Caenorhabditis elegans* embryo.

## CYK-4 protein: a brief introduction

**Figure 1.**
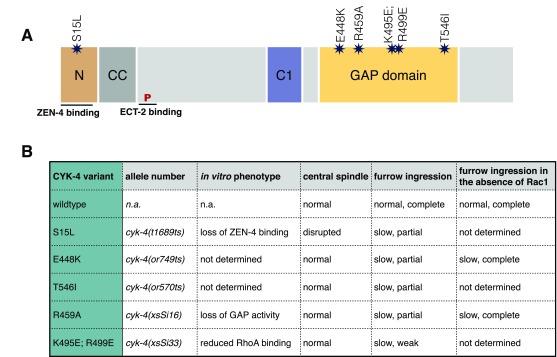
Domain structure and mutational analysis of CYK-4. ( **A**) Schematic of the CYK-4 protein and its constituent domains indicating the positions and nature of the mutations studied in *Caenorhabditis elegans*. ( **B**) Table summarizing phenotypic details of CYK-4 mutations studied in *C. elegans*. Rac1 depletion permits complete furrowing in E448K mutants, leading to a proposal that CYK-4 functions to inactivate Rac1 during cytokinesis. In this review, we discuss an alternative interpretation of the results.

CYK-4, so named in *C. elegans*, comprises an N-terminal coiled coil, a central membrane-binding C1 domain, and a C-terminal RhoGAP domain ( Figure 1A). It is known as MgcRacGAP in mammalian cells and as RacGAP50C and *tumbleweed* in *Drosophila.* CYK-4 exists as a heterotetramer with ZEN-4 (MKLP1 in mammals and *pavarotti* in *Drosophila*) ^16^. This multimeric complex, dubbed centralspindlin, accumulates and drives bundling of antiparallel microtubules in the anaphase spindle midzone ^17– 22^. It can also be detected in small quantities at the equatorial plasma membrane in anaphase ^21, 23– 25^, where it is well situated to act as a RhoA activator.

Prior to anaphase, the activity of the cytokinetic RhoGEF ECT-2 is suppressed by autoinhibition ^26^ and Cdk1 phosphorylation ^27^. Curiously, the RhoGAP protein CYK-4, which ordinarily would be predicted to turn a GTPase “off”, interacts with ECT-2 via their respective N-termini in a phospho-regulated manner ^12^. This interaction occurs in a wide variety of contexts, assays, and organisms and recruits the GEF to the midzone ^12, 14, 28– 31^. Centralspindlin contributes to localization or activation of ECT-2 (or both) at the plasma membrane, where this RhoGEF primarily acts ^27^. Barring the noteworthy *C. elegans* embryo (discussed in detail below), depleting CYK-4 in animal cells blocks the formation of a cleavage furrow, suggesting that it positively regulates RhoA activity ^2, 11, 12, 14, 28, 32^. Does the CYK-4 GAP domain contribute positively or negatively to RhoA regulation? While the case of CYK-4 is pertinent to cytokinesis and GTPase regulation in diverse systems, the function of this protein has been most extensively analyzed in one-cell *C. elegans* embryos.

## Two pathways for RhoA activation in *C. elegans*

The one-cell *C. elegans* embryo, despite being a powerful system for studying the cell biology and genetics of cytokinesis, has a complication not observed in other systems. RhoA activation during cytokinesis in the early *C. elegans* embryo, unlike cultured human cells and *Drosophila* cells, depends on the combined action of centralspindlin and a second pathway. This second pathway involves a distinct activator of ECT-2 which has a distinct means for spatial regulation of its activity. While this can be confounding for *cyk-4* analyses, it is not insurmountable. This second pathway can be isolated from the centralspindlin-directed pathway by attenuation of spindle pulling forces ^11^, severing the central spindle with a laser ^33^, and altering spindle position ^34^. Most recently, a specific molecular component of this second pathway, NOP-1, was identified ^2^. Because of their overlapping functions, in order to delineate the role of CYK-4 in cleavage furrow formation, the alternate furrow induction pathway must be eliminated. The simplest means to do so is by inactivating NOP-1.

*nop-1* is a non-essential, poorly conserved, nematode-specific gene. Nevertheless, it promotes RhoA activation during polarization of the *C. elegans* zygote ^2, 35^. NOP-1–deficient embryos complete cytokinesis with wild-type kinetics, but embryos defective in both NOP-1 and CYK-4—by RNA interference (RNAi) or any of the point mutants discussed below—do not initiate cleavage furrows ^2, 36^. Imaging with a RhoA biosensor confirms that CYK-4 and NOP-1 coordinately promote RhoA activation upstream of ECT-2 during cytokinesis and that CYK-4 plays the more prominent role ^2^.

The NOP-1 pathway appears capable of directing global RhoA activation in the embryo. However, astral microtubules limit the sites of active RhoA accumulation to the anterior of the zygote during polarization and to broad regions at the equator and the most anterior part of the zygote during anaphase. The NOP-1 and centralspindlin pathways can be readily spatially separated by manipulating the spindle to position it at the posterior of the embryo ^2, 34^. At anaphase, an embryo, so modified, produces an anterior furrow that is NOP-1–dependent and a posterior furrow that is centralspindlin-dependent.

Although the biochemistry of NOP-1 activation of ECT-2/RhoA is unknown, NOP-1–deficient *C. elegans* embryos resemble other eukaryotic cells in that cleavage furrow formation, and ingression are completely dependent upon CYK-4, thus providing a straightforward way to analyze the function of the latter.

## Genetic dissection of the CYK-4 GAP domain in *C. elegans* embryos

### CYK-4 GAP domain mutants

While there is compelling evidence that CYK-4 plays a role in RhoA activation, the role of its GAP domain remains contentious. The isolated GAP domain of CYK-4 promotes GTP hydrolysis *in vitro* by Rho family members but has much stronger activity toward Rho GTPases Rac and Cdc42 when compared with RhoA ^22, 37, 38^. Importantly, however, Rac1 and Cdc42 are dispensable for cytokinesis in a wide variety of contexts ^36, 39– 41^. Thus, do these *in vitro* assays properly reflect the function of full-length CYK-4 during cytokinesis *in vivo*? Does it function as a conventional GAP that inactivates Rac or RhoA or both, or does it participate, unexpectedly, in activating RhoA via ECT-2, or a combination thereof? **

CYK-4 was first implicated in cytokinesis on the basis of a temperature-sensitive lethal mutation— *cyk-4(t1689ts)*—in *C. elegans* encoding an S15L substitution ( Figure 1A) in the N-terminus of CYK-4 that disrupts its interaction with ZEN-4 ^16, 42^. Subsequently, Canman and colleagues isolated two additional mutations— *cyk-4(or749ts)*, yielding CYK-4 ^E448K^, and *cyk-4(or570ts)*, yielding CYK-4 ^T546I^—and both substitutions map to the GAP domain of CYK-4 ^43^. However, these mutations are not in the active site, and the biochemical nature of these alleles has not been characterized. Therefore, it is risky to assume that these mutants are solely defective in CYK-4 GAP activity. Indeed, CYK-4 ^E448K^ exhibits a defect in membrane localization in the gonad ^36^. However, because the mutants assemble normal central spindles, they cannot be entirely unfolded at the restrictive temperature. More recently, a mutant targeting the conserved catalytic arginine in the GAP domain was generated (R459A), as was a variant where two surface residues were mutated (K495E and R499E) to block CYK-4 interaction with Rho family GTPases ^36^. None of the *cyk-4* mutations mentioned here abolishes furrow formation in otherwise wild-type embryos; furrows ingress partially and regress ( Figure 1).

The debate regarding CYK-4 GAP domain function was initiated by the discovery that depleting the GTPase Rac1 (CED-10 in *C. elegans*) in CYK-4 ^E448K^ embryos frequently results in complete ingression of the furrow ^43^. This was interpreted to mean that the primary function of the CYK-4 GAP domain during wild-type cytokinesis is to inactivate Rac1/CED-10 ^43, 44^. The experimental result ( Figure 1B) is uncontested and has been confirmed by using either RNAi ^36, 43^ or loss-of-function alleles ^44, 45^ to inactivate Rac1/CED-10. However, there is significant disagreement regarding interpretation of the results and the underlying mechanism of CYK-4 GAP function.

### Redundant furrow induction pathways obscure CYK-4 function

The presence of a parallel pathway for furrow induction contributes to the difficulty in interpreting results concerning the role of the CYK-4 GAP domain. If a given GTPase is the sole, relevant target of the CYK-4 GAP domain during cytokinesis, then its loss should reverse the defects of CYK-4 GAP mutants in a variety of genetic contexts. However, inactivation of Rac1/CED-10 rescues furrow ingression in GAP domain mutants (E448K, R459A, and T546I) ^36, 43– 45^ only in the presence of NOP-1 ^36^. In NOP-1 mutant embryos, which are cytokinesis proficient, mutations in the GAP domain of CYK-4 block furrow formation altogether and the activity of Rac1/CED- 10 has no bearing on this behavior ( Table 1) ^36^. If mutations in the GAP domain of CYK-4 indeed cause Rac1/CED 10 hyperactivation, then inactivation of Rac1/CED-10 should correct this defect regardless of the presence or absence of NOP-1.

**Table 1.** Summary of available data for RhoA and Rac1 effector accumulation and furrowing phenotypes during cytokinesis in two key *Caenorhabditis elegans cyk-4* GTPase-activating protein mutants.

CYK-4 variant	RhoA effector accumulation at furrow	Rac1 effector hyperaccumulation at furrow	Furrow ingression	Furrow ingression in the absence of Rac1	Furrow ingression in the absence of NOP-1	Furrow ingression in the absence of NOP-1 and Rac1
Wild-type	+++	Absent	Normal, complete	Normal, complete	Normal, complete	Normal, complete
E448K	*	Not determined	Slow, partial	Slow, complete	Absent	Absent
R459A	+	Not determined	Slow, partial	Slow, complete	Absent	Absent

*Conflicting data; see the “cyk-4 GAP mutants have multiple defects” section. +++ Wild-type accumulation of myosin II, RhoA biosensor. + Reduced accumulation of myosin II, RhoA biosensor.

In addition to NOP-1 inactivation, there are other ways in which the centralspindlin-dependent cytokinetic furrow in *C. elegans* embryos can be isolated from the alternative pathway (see “CYK-4 protein: a brief introduction”). In each case, the CYK-4 ^E448K^ mutation prevents centralspindlin-dependent furrow formation, and, again, inactivation of Rac1/CED-10 does not restore furrowing ^45^. Stated another way, loss of Rac1/CED-10 reverses the furrowing defect in CYK-4 GAP mutants only when the centralspindlin-independent pathway for furrow formation is active (that is, when an alternative pool of locally activated RhoA is available). Strikingly, in their recent publication, Canman and colleagues did not use any of these approaches to test whether Rac1/CED-10 is a direct target of CYK-4 GAP activity ^44^.

### *cyk-4* GAP mutants have multiple defects

To analyze CYK-4 GAP domain function using *cyk-4* mutants in a meaningful way, all aspects of a mutant phenotype must be considered. In addition to being incomplete, furrows in embryos with *cyk-4* GAP domain mutations have slower ingression kinetics and weakly accumulate active RhoA and its effectors. Although Rac1/CED-10 depletion in *cyk-4* GAP mutant embryos permits the completion of furrow ingression, it is unable to restore the other two phenotypes ^44, 45^. If Rac1/CED-10 was the primary relevant target of CYK-4, embryos deficient in both Rac1/CED-10 and CYK-4 GAP activity would be predicted to resemble Rac1/CED-10–deficient embryos (which closely resemble wild-type embryos).

**Figure 2.**
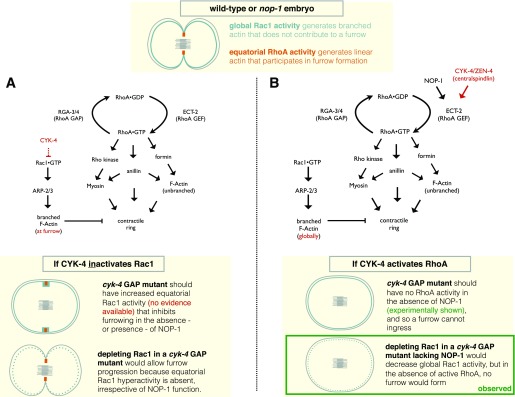
Proposed models for function of the CYK-4 GTPase-activating protein (GAP) domain in *Caenorhabditis elegans* with predicted phenotypes. At the top, a diagram depicting Rac1 and RhoA activities in a dividing wild-type or *nop-1* mutant embryo. ( **A**) Genetic pathway and schematic model for cytokinetic furrow formation in *C. elegans* where CYK-4 solely functions to inactivate Rac1, as described by 44. In this model, ECT-2 is not subject to activation. However, as discussed in the text, available experimental evidence does not support this model. ( **B**) Genetic pathway and schematic model for cytokinetic furrow formation in *C. elegans* where CYK-4 and NOP-1 function in parallel upstream of ECT-2 in RhoA activation. The diagrams at the bottom of ( **A**) and ( **B**) reflect scenarios where NOP-1 is absent.

Canman and colleagues recently suggested that mutations in the GAP domain of CYK-4 do not impair myosin accumulation ( Table 1, asterisk) ^44^, despite Canman and colleagues’ earlier work demonstrating a defect in myosin at the furrow tip in *cyk-4* mutant embryos (see Figure S3 in 43). A variety of mutations in the CYK-4 GAP domain impair the accumulation of myosin, actin, anillin, and a RhoA biosensor ( Table 1, asterisk) ^2, 36, 45^. Again, these defects are not corrected by depleting Rac1. This is particularly dramatic in NOP-1–deficient embryos where myosin and RhoA activation during cytokinesis are entirely dependent upon centralspindlin ^2, 36^. These results cannot be explained if the CYK-4 GAP domain acts on Rac1/CED-10. Rac1/CED-10 activates the Arp-2/3 complex ^46, 47^, a nucleator of branched actin filaments ^48^. If CYK-4 GAP mutations prevent Rac1 inactivation, hyperaccumulation of Rac1/CED-10 effectors or an increase in branched actin on the membrane would be expected in these mutants. But, in fact, *cyk-4* mutations result in a reduction in actin accumulation ^45^. Critically, there is no evidence that mutations in the GAP domain of CYK-4 cause ectopic Rac1/CED-10 activation in the *C. elegans* embryo ( Table 1 and Figure 2A). Collectively, the available data do not support models in which Rac1/CED-10 is the primary relevant target of CYK-4 GAP activity during cytokinetic furrowing.

### An indirect role for Rac1/CED-10 in furrow formation

Why does loss of Rac1/CED-10 allow CYK-4 GAP mutants to complete furrow ingression? Inactivation of Rac1/CED-10 partially rescues the cytokinesis defect resulting from selected mutations in CYK-4 and only in the presence of a redundant furrow induction pathway. The extremely specific situations in which restoration to full ingression is observed strongly support that this behavior is mediated by bypass suppression. That is, instead of correcting the primary defect resulting from the mutation in CYK-4, the absence of Rac1/CED-10 attenuates the requirement for RhoA activation in furrow ingression. Depletion of ARX-2, a subunit of the ARP-2/3 complex, has a similar effect ^43, 45^. Inactivation of Rac1/CED-10 or ARX-2 also partially remedies the cytokinesis defects caused by weak mutations in ECT-2 ^45^. While RhoA activates formins to generate linear actin filaments at the furrow, the anterior cortex of the embryo is enriched in ARP-2/3 nucleated branched actin ^49– 52^. Depleting ARX-2 or Rac1/CED-10 induces cortical instability ^45, 53^, which may allow the weak, NOP-1–dependent furrow to ingress further because the cortex is now more pliant. Alternatively, or in addition, depolymerization of this pool of branched F-actin may allow increased actin polymerization in the furrow because of a reduction in competition for actin monomer ^54^. In either case, the suppression is due to enhancement of the centralspindlin-independent pathway.

Canman and colleagues have suggested that if Rac1/CED-10 depletion generally promotes ring constriction, then Rac1/CED-10 depletion should also be able to rescue weak contractile rings resulting from temperature-sensitive mutations of the formin CYK-1 and non-muscle myosin II (NMY-2) at intermediate temperatures ^44^, neither of which is observed. However, as both furrowing pathways are active in this experiment and both strictly require formin and myosin for furrow formation, it is not entirely surprising that Rac1/CED-10 depletion does not compensate when major structural components of the contractile ring are crippled. Direct visualization of the sites of accumulation of branched actin in *cyk-4* mutant embryos and assays for the effects of Rac1/CED-10 and the Arp2/3 complex on cortical stability during cytokinesis will help better understand these results.

### An alternative model for CYK-4 GAP domain function

The data discussed above point to a compelling, albeit counterintuitive, role for CYK-4 in the activation of RhoA during cytokinesis ( Figure 2B). As stated previously, in many model systems, the CYK-4 N-terminus directly interacts with the RhoGEF ECT-2 and is responsible for the relief of autoinhibition and can contribute to the recruitment of the latter to the division plane. Where analyzed, depletion of CYK-4 results in weak or no furrows. This is also the case in *C. elegans* CYK-4 GAP mutants ( Figure 1). Importantly, as stated in the “ *cyk-4* GAP mutants have multiple defects” section above, these CYK-4 mutations result in reduced accumulation of RhoA effectors at the furrow. Disabling parallel furrow induction pathways (see “CYK-4 protein: a brief introduction”) in CYK-4 GAP mutants abolishes the residual accumulation of active RhoA and its targets.

As the above results suggest, if CYK-4 GAP mutants are truly defective in activating RhoA, then experimentally increasing active RhoA levels should restore full RhoA function and cytokinetic furrowing. Importantly, this should occur even in the absence of a redundant furrowing pathway. This prediction was tested in two ways. As mentioned previously, CYK-4 GAP mutants do not furrow in the absence of NOP-1. Remarkably, depletion of RGA-3/4, a conventional RhoGAP that switches off RhoA ^55, 56^, results in wild-type cytokinetic furrowing in CYK-4 GAP mutants in the presence and absence of NOP-1 ^36^. Increasing RhoA activity using an activated allele of the RhoGEF ECT-2 has the same striking effect ^36^. These results together support the hypothesis that CYK-4 GAP domain participates in RhoA activation. The results described herein are compiled in poster format viewable on figshare ( 10.6084/m9.figshare.5325619).

In addition to interacting via their N-termini, an association between the CYK-4 GAP and the ECT-2 GEF domain has also been reported ^36^. The function of this interaction remains to be elucidated. The GAP domain may facilitate relief of ECT-2 autoinhibition and participate in a ternary complex with RhoA to fully activate the GTPase. While such a model may explain results from *C. elegans* embryos, CYK-4 function could be highly context-dependent and may vary with diverse cellular parameters.

## Context dependence: other experimental systems

Thus far, we have discussed results concerning the GAP domain of CYK-4 in *C. elegans* embryos, the system that has been studied in the most detail. The conservation of the GAP domain suggests that GAP activity is important for the organism; however, this conservation does not imply that the activity is critical in all cell types. Indeed, while the GAP activity of CYK-4 is essential for cytokinesis in early blastomeres, it is dispensable later in embryogenesis, except in the germline ^36^. The mechanisms that are required for RhoA activation employed by a given cell likely depend on features such as cell-cell or cell-substrate adhesion, cell size, spindle size, spindle–to–plasma membrane distance, and relative abundance of proteins. There are a number of other examples where the genetic requirements for cytokinesis depend on the cellular or tissue context ^57, 58^. Experimentally, using optogenetics to control RhoA activity, cleavage furrow ingression can be dramatically slowed by polar or global activation of RhoA ^8^.

The requirement for the GAP domain of CYK-4 and its orthologs has been studied in a number of other contexts. While CYK-4 protein is required in all cells examined to date, the requirement for the GAP domain and its catalytic activity varies. In chicken B cells, the CYK-4 GAP domain, but not the catalytic arginine, is required for cytokinesis ^59^, and mutations in the GAP domain strongly impair furrow formation in *Drosophila* ectoderm ^60^. These results are largely consistent with a role for the GAP domain in promoting RhoA activation.

The role of CYK-4 GAP activity has been investigated in some detail in HeLa cells. In these cells, catalytically inactive CYK-4 displays a range of phenotypes, including many cells with a late cytokinesis defect in which cells ingress with apparently normal kinetics but fail to undergo abscission ^38^. Inactivation of CYK-4 GAP activity results in a dramatic delocalization of the RhoA effector anillin. In addition, there is increased cell spreading and adhesion to the substrate, both in the midzone where CYK-4 concentrates and in the cell periphery. Cytokinesis failure in these cells could be suppressed by depletion of Rac1 or selected effectors. These results are consistent with a model in which CYK-4 targets Rac1 in this context, although indirect effects involving GTPase crosstalk are possible ^61^.

In *Xenopus* embryos, CYK-4 is also essential for RhoA activation. A useful feature of this system is that cytokinetic RhoA activation can be readily measured with a biosensor that reveals a distinct “Rho zone” during cytokinesis. When the GAP domain is deleted, RhoA activation occurs, but the Rho zone appears to move as a wave in the plane of the membrane. Conversely, inactivation of GAP activity results in a broadening of the Rho zone during cytokinesis ^62^. Thus, unlike the case in *C. elegans* embryos, activation of RhoA in *Xenopus* does not strictly depend on the GAP activity or its RhoA-binding activity. The expansion seen in the catalytically inactive mutant in *Xenopus* is consistent with a conventional view of GAP function in which CYK-4 acts to inactivate RhoA, pointing to a requirement for flux in maintaining the size of the Rho zone. If that was the sole role of the GAP domain, the GAP domain deletion mutant would be expected to behave similarly. Rather, the mobility of the Rho zone in the deletion mutant indicates that GAP domain plays a role in anchoring the Rho zone. Evidence from *C. elegans* indicates that the RhoA GAP domain associates with active RhoA ^36^. These two findings in *Xenopus* also fit a model in which binding of CYK-4 to active RhoA promotes positive feedback through ECT-2 recruitment. Mutation of the active site of CYK-4 GAP could induce expansion of the Rho zone by enhancing positive feedback, as the GAP-deficient mutant binds more strongly to active RhoA than wild-type CYK-4. The GAP domain deletion mutant obviously lacks this functionality which could allow for the observed movement of the Rho zone.

## Summary

The results described here reveal that cytokinesis in metazoa is driven by a contractile ring that assembles in response to local RhoA activation at the equatorial cortex during anaphase. This is one of the near-universal features of the process; other aspects vary in different cell types and organisms. The extent of RhoA activation required to divide a cell will depend considerably on its context. Division of a large embryonic blastomere is unlikely to be identical to that of a small somatic cell of the same animal. Similarly, we have described that changes to the properties of the overall cell cortex of the large blastomere can alter the requirements for cytokinesis.

Nevertheless, the proteins that mediate RhoA activation are well conserved among metazoa. Many regulatory features are conserved, but the extent to which they are required is more variable. There is ample evidence that caution must be used when extrapolating from one system to another. Here, we have focused on CYK-4 during cytokinesis in the early *C. elegans* embryo. In this system, the available evidence reveals that the GAP activity of CYK-4 promotes RhoA activation. The underlying mechanism will be fascinating to dissect.
